# Compensatory patterns of collateral flow in stroke patients with unilateral and bilateral carotid stenosis

**DOI:** 10.1186/s12883-016-0560-0

**Published:** 2016-03-18

**Authors:** Hui Fang, Bo Song, Bo Cheng, Ka Sing Wong, Yu Ming Xu, Stella Sin Yee Ho, Xiang Yan Chen

**Affiliations:** Department of Neurology, the First Affiliated Hospital of Zhengzhou University, No.1 Jianshe East Road, Erqi District, Zhengzhou, Henan PR China; Department of Medicine and Therapeutics, Faculty of Medicine, Prince of Wales Hospital, The Chinese University of Hong Kong, Shatin, New Territories Hong Kong; Department of Imaging and Interventional Radiology, Faculty of Medicine, Prince of Wales Hospital, The Chinese University of Hong Kong, Shatin, New Territories Hong Kong

**Keywords:** Collateral circulation, Carotid stenosis, Stroke, Ultrasonography

## Abstract

**Background:**

Collateral pathways are important in maintaining adequate cerebral blood flow in patients with carotid stenosis. We aimed to evaluate the hemodynamic patterns in relation to carotid stenosis in acute stroke patients.

**Methods:**

Consecutive 586 stroke patients in a hospital based cohort were included in the present study. Carotid duplex was performed to identify patients with absolute minimal diameter reductions of 50 % or greater in their internal carotid arteries (ICAs). Color velocity imaging quantification ultrasound (CVIQ) was used to measure extracranial arterial blood flow volume (BFV) in bilateral common carotid arteries (CCAs) and bilateral vertebral arteries (VAs). The absolute values of BFV and the ratios were compared between patients with and without ICA stenosis.

**Results:**

Among 586 acute ischemic stroke patients (mean age: 67.5 ± 12.4y), ICA stenosis was detected in 112 patients (19.1 %), including unilateral ICA stenosis in 81 patients (13.8 %) and bilateral ICA stenosis in 31 patients (5.3 %). Among patients with unilateral ICA stenosis, the BFV in contralateral CCA was significantly higher than that in ipsilateral CCA (325.5 ± 99.8 mL/min vs. 242.2 ± 112.2 mL/min, *P* < 0.001). Among patients with bilateral ICA stenosis, the sum of BFV in bilateral VAs accounted for 22 % of the whole cerebral blood flow, which was significantly higher than that in those without ICA stenosis (14.8 %, *P* < 0.001) or with unilateral ICA stenosis (16.9 %, *P* = 0.007).

**Conclusions:**

In patients with unilateral carotid stenosis, contralateral carotid blood flow increases to compensate decreased blood flow, while posterior circulation may compensate for the decreased brain perfusion in those with bilateral carotid stenosis.

## Background

According to the results from large randomized trials, carotid endarterectomy has become the accepted standard treatment of choice for patients with recently symptomatic carotid stenosis, and selected patients can benefit from revascularization with endarterectomy or stenting [1]. However, limited information has been obtained regarding optimal selection of revascularization candidates. The current approach to patient selection is primarily based on anatomical identification of luminal stenosis, without taking into account compensatory mechanisms, poststenotic flow, downstream perfusion status, and presence of collateral circulation [2–4], which thus may be insufficient and misleading. It is well recognized that simply an anatomically luminal stenosis might not always lead to impaired cerebral hemodynamics and hypoperfusion in the distal cerebral circulation [5, 6]. Therefore, it is necessary to develop imaging techniques that can be used to evaluate the downstream hemodynamics and cerebral perfusion of extracranial steno-occlusive disease [7].

Color velocity imaging quantification (CVIQ) ultrasound has been proved to be a noninvasive technique that can reveal extracranial arterial blood flow volume, which is an indicator of cerebral perfusion [8–12]. Our previous studies demonstrated that poststroke extracranial arterial blood flow volume estimated by CVIQ is an independent predictor of functional outcome and recurrent stroke [11, 12]. In addition, it is important to assess the contribution of the contralateral ICA, the ipsilateral ECA, and the vertebral arteries to the collateral pathways in patients with symptomatic ICA stenosis [13–15]. The quantitative measurement of carotid and vertebral blood flow volume (BFV), which has been suggested as an estimate of total cerebral BFV is clinically of great value in evaluating the presence of collateral pathways and hemodynamic conditions in patients with carotid stenosis [16–18]. Furthermore, compared with other quantitative examinations to determine cerebral perfusion as MRI perfusion, CT perfusion, Single Photon Emission Computed Tomography (SPECT), CVIQ is a noninvasive, easy to access, economic, and free of any known harm techniques which requires no contrast agent. In the present study, we evaluated the hemodynamic patterns in acute stroke patients with unilateral and bilateral internal carotid artery (ICA) stenosis by using CVIQ ultrasound.

## Methods

### Patients

It was a hospital based cohort study including consecutive stroke patients admitted to the Prince of Wales Hospital (PWH) from January 1, 1998 to December 31, 2000. The study was approved by the Joint Chinese University of Hong Kong – New Territories East Cluster Clinical Research Ethics Committee (The Joint CUHK-NTEC CREC). Informed written consent was obtained from each patient before blood flow volume measurement. The patients were recruited according to the inclusion criteria: (1) Sudden onset of neurological symptoms that persisted for ≥24 h based on the World Health Organization criteria; (2) Diagnosis confirmed by a CT scan of the head that excluded hemorrhage or other alternative diagnosis. Patients with cardioembolic stroke according to the Trial of ORG 10172 in Acute Stroke Treatment (TOAST) criteria and those who were clinically unstable for transfer to the ultrasound vascular laboratory for the study were excluded.

### Data acquisition

The assessment of carotid stenosis and measurement of BFV was performed as described previously [8, 9, 11, 12]. A 7.5-MHz linear transducer of a Philips SD800 ultrasound unit (Best, the Netherlands) which has a standard feature of duplex Doppler imaging as well as a color velocity imaging quantification (CVIQ) software for blood flow volume measurement was used. Carotid duplex was performed to determine the presence of ICA stenosis (≥50 %). Carotid stenosis was defined as the ratio of the peak systolic velocity at the internal carotid artery to that at the ipsilateral common carotid artery greater than 1.5 or direct measurement of the stenosis greater than 50 % on the static image if the true and residual lumen could be clearly depicted. The ipsilateral-to-contralateral CCA blood flow volume ratio was defined as the ratio of the BFV in the ipsilateral CCA to that in the contralateral CCA.

CVIQ technique was performed to measure BFV in bilateral common carotid arteries (CCAs) and bilateral vertebral arteries (VAs). The average of 3 repeated measurements was taken as the BFV of an individual artery. The intra- and interobserver reproducibility was tested by two operators utilizing the standardized technique. The intraclass correlation coefficient for interobserver reproducibility between operators A and B was 0.75 and those for intraobserver reproducibility of operators A and B were 0.88 and 0.93 (*p* < 0.001), respectively.

According to the presence of ≥50 % carotid stenosis, patients were divided into three groups: patients without carotid stenosis as control group, those with unilateral carotid stenosis and those with bilateral carotid stenosis.

### Statistical analysis

All values were indicated as mean ± standard deviation, and tested for normal distribution by the Kolmogorov-Smirnov procedure. Two-tailed Student *t* test was performed to compare the values with normal distribution. *P* value of less than 0.05 was considered to be significant. Spearman correlation was used to assess the relationship between degree of artery stenosis and blood flow volume. The absolute values and the ratios of BFV in different arteries were compared between the patients with unilateral and bilateral ICA stenosis. All analyses were performed with SPSS.

## Results

### Baseline characteristics

During the study period, 586 acute ischemic stroke patients were recruited (mean age, 67.5 ± 12.4y). Unilateral ICA stenosis was detected in 81 (13.8 %) patients and bilateral ICA stenosis in 31 (5.3 %) patients by using carotid duplex with 1 week after stroke onset. The baseline characteristics of stroke patients were summarized in Table 1. Compared with patients without carotid stenosis, patients with unilateral or bilateral carotid stenosis were older (*P* = 0.002), and had a higher rate of male (*P* = 0.008), hypertension (*P* = 0.002) and previous cerebral vascular diseases (*p* = 0.035). However, stroke patients with unilateral and bilateral carotid stenosis had comparable prevalence of age and risk factors (Table 2).

**Table 1:** Baseline Characteristics of the whole stroke patients and comparisons between patients with and without ICA stenosis

Baseline characteristics	Total *N* = 586	Patients without ICA stenosis *n* = 474	Patients with ICA stenosis *n* = 112	*P* value
Male sex, %	316, 53.9 %	243, 51.3 %	73, 65.2 %	0.008
Mean age, y	67.5 ± 12.4	66.7 ± 12.8	70.7 ± 9.7	0.002
Hypertension, %	309, 52.7 %	235, 49.6 %	74, 66.1 %	0.002
Diabetes Mellitus, %	181, 30.9 %	139, 29.3 %	42, 37.5 %	0.092
Hyperlipidemia, %	192, 32.8 %	154, 32.5 %	38, 33.9 %	0.783
Smoking, %	162, 27.6 %	125, 26.4 %	37, 33 %	0.156
Ischemic Heart Disease, %	227, 38.7 %	175, 36.9 %	52, 46.4 %	0.063
Previous Stroke or TIA, %	148, 25.3 %	111, 23.4 %	37, 33 %	0.035
Blood Flow Volume	727.2 ± 188.3	738.3 ± 189.6	680 ± 175.7	0.003
Bilateral CCAs	615.8 ± 167.6	629.2 ± 167.4	559.1 ± 156.9	<0.001
Bilateral VAs	111.8 ± 55.8	109.1 ± 52.5	123.2 ± 67.3	0.016
Bilateral CCA%	84.6 ± 6.8	85.2 ± 5.9	82.2 ± 9.3	0.001
Bilateral VA%	15.5 ± 6.6	14.8 ± 5.9	18.3 ± 8.6	<0.001

**Table 2:** Comparisons of clinical features between stroke patients with unilateral and bilateral ICA stenosis

	Patients with unilateral ICA stenosis *n* = 81	Patients with bilateral ICA stenosis *n* = 31	*P*-value
Male sex, %	53, 65.4 %	20, 64.5 %	0.927
Mean age, y	70.6 ± 9.7	71.2 ± 9.8	0.744
Hypertension, %	56, 69.1 %	18, 58.1 %	0.268
Diabetes Mellitus, %	31, 38.3 %	11, 35.5 %	0.785
Hyperlipidemia, %	30, 37 %	8, 25.8 %	0.379
Smoking, %	27, 33.3 %	10, 32.3 %	0.914
Ischemic Heart Disease, %	39, 48.1 %	13, 41.9 %	0.555
Previous Stroke or TIA, %	28, 34.6 %	9, 29 %	0.577
Patients with carotid stenosis >70 %, %	43, 53.1 %	22, 71 %	0.065
Blood Flow Volume	681.7 ± 175.5	675.6 ± 179.1	0.872
BA	114.3 ± 64.4	146.2 ± 70.4	0.024
BA%	16.8 ± 8.1	22 ± 8.9	0.004
Bilateral CCAs	567.3 ± 160.3	537.4 ± 147.9	0.369
Bilateral CCAs%	83.2 ± 8.1	79.6 ± 11.8	0.065

### Hemodynamic data

Comparisons of hemodynamic data between stroke patients without ICA stenosis and with unilateral or bilateral ICA stenosis were described in Table 3. Compared with those without carotid stenosis (738.3 ± 189.6 ml/min), the total BFV decreased in patients with unilateral carotid stenosis (681.7 ± 175.5 ml/min; *p* = 0.012) and tended to decrease in those with bilateral carotid stenosis (675.6 ± 179.1 ml/min; *p* = 0.074). The sum of BFV in bilateral VAs increased but did not reach statistical significance compared with patients without ICA stenosis (114.3 ± 64.4 ml/min vs. 109.1 ± 52.5 ml/min, *P* = 0.421). However, the percentage of BFV in bilateral VAs accounted for almost 16.9 % of the whole brain BFV, which was significantly higher than 14.8 % in those without ICA stenosis (*P* = 0.036).

**Table 3:** Comparisons of hemodynamic data between stroke patients without ICA stenosis and those with unilateral or bilateral ICA stenosis

	Patients without ICA stenosis *n* = 474	Patient with unilateral ICA stenosis *n* = 81	*P* value*	Patient with bilateral ICA stenosis *n* = 31	*P* value**
Total BFV	738.3 ± 189.6	681.7 ± 175.5	0.012	675.6 ± 179.1	0.074
Bilateral CCA	629.2 ± 167.4	567.3 ± 160.3	0.002	537.4 ± 147.9	0.003
Bilateral CCA%	85.0 ± 6.0	83.0 ± 8.0	0.036	79.5 ± 11.8	0.013
BFV in bilateral VAs	109.1 ± 52.5	114.3 ± 64.4	0.421	146.2 ± 70.4	<0.001
% BFV in bilateral VAs	14.8 ± 5.9	16.9 ± 8.1	0.036	22.0 ± 8.9	<0.001

*indicates the comparison between patients with unilateral ICA stenosis and patients without ICA stenosis

**indicates the comparison between patients with bilateral ICA stenosis and patients without ICA stenosis

**Fig. 1:**
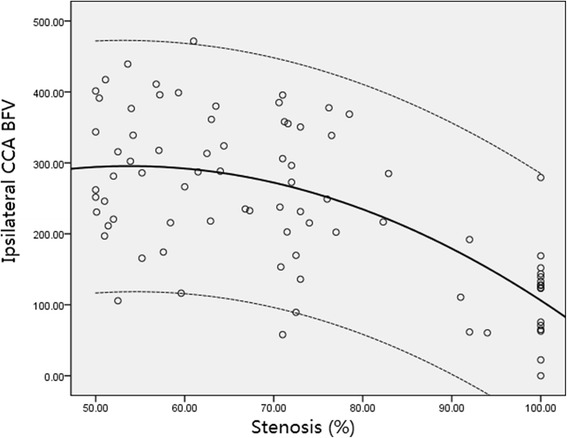
A decrease in ipsilateral CCA blood flow is significantly associated with increasing percentage

**Fig. 2:**
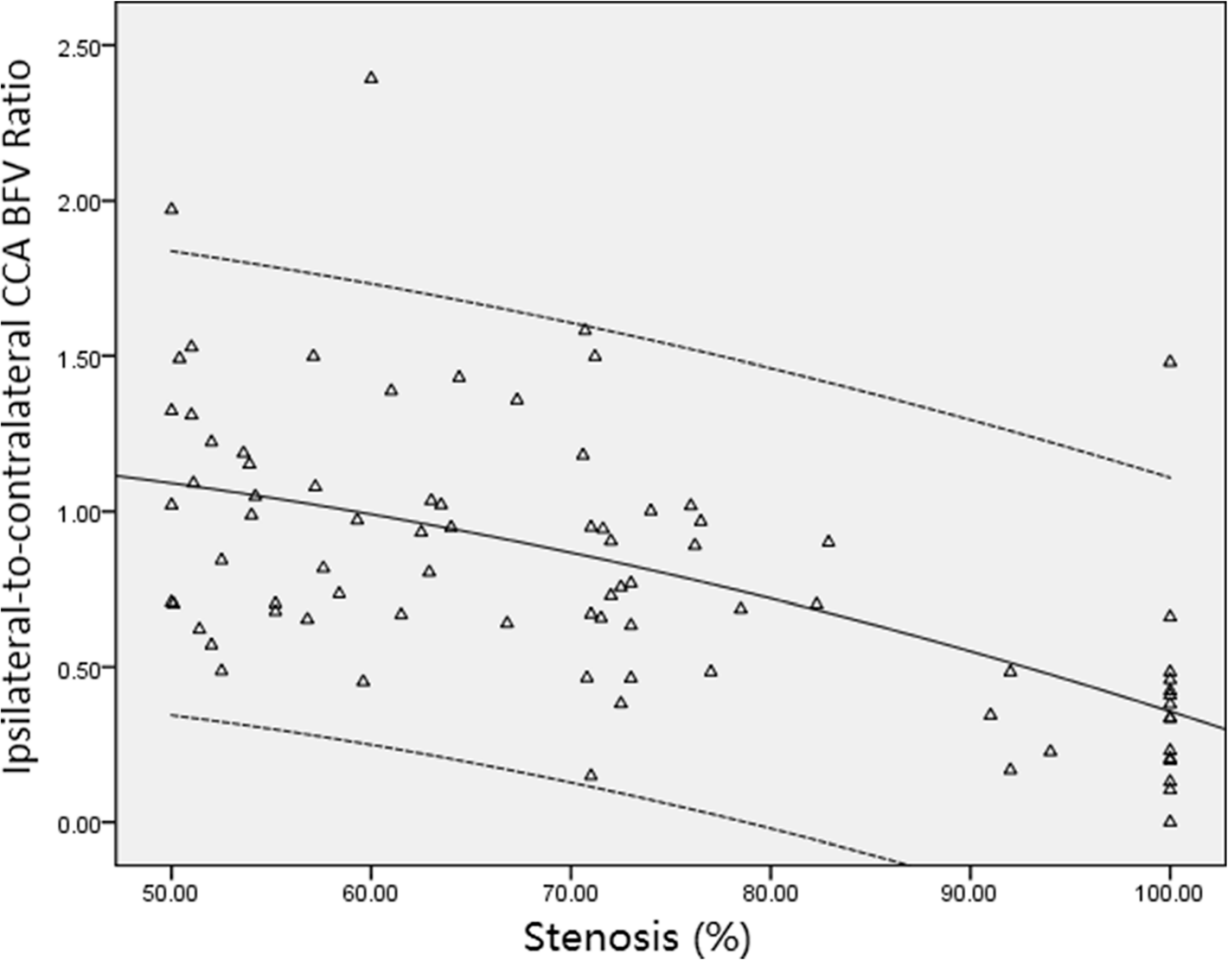
A decrease in ipsilateral-to-contralateral CCA BFV Ratio is significantly associated with increasing percentage

Among stroke patients with unilateral ICA stenosis, the absolute values of BFV in ipsilateral CCA or the ratios decreased with the increasing severity of ICA stenosis, as shown in Figs. 1 and 2. The absolute values of BFV in contralateral CCA, as well as the ratios of contralateral CCA BFV, was significantly higher than that in ipsilateral CCA (325.5 ± 99.8 mL/min vs. 242.2 ± 112.2 mL/min, *P* < 0.001; 48.5 ± 12.2 % vs. 34.7 ± 12.5 %, *P* < 0.001) (Table 4). However, there were 25 (30.9 %) patients with significant increase in BFV in ipsilateral CCA (323.8 ± 75.4), implying collateral pathways from inpsilateral ECA artery.

**Table 4:** Comparisons of BFV in ipsilateral and contralateral CCA among stroke patients with unilateral ICA stenosis

	Ipsilateral CCA	Contralateral CCA	*P* value
BFV	242.2 ± 112.2	325.5 ± 99.8	<0.001
%BFV	34.7 ± 12.5	48.5 ± 12.2	<0.001

Compared to stroke patients without ICA stenosis, the absolute values of bilateral CCA BFV in patients with bilateral ICA stenosis and the ratios decreased significantly (567.3 ± 160.3 vs. 629.2 ± 167.4, *P* = 0.002; 83.0 ± 8.0 % vs. 85.0 ± 6.0; *P* = 0.036). The sum of BFV in bilateral VAs increased significantly compared with patients without ICA stenosis (146.2 ± 70.4 ml/min vs. 109.1 ± 52.5 ml/min, *P* < 0.001) and accounted for almost 22 % of the whole brain BFV, which was significantly higher than 14.8 % in those without ICA stenosis (*P* < 0.001).

## Discussion

In patients with unilateral carotid artery stenosis, evidence is accumulating to suggest that compromised hemodynamic brain state is associated with an increased risk of stroke than if they have a normal brain perfusion [19–21]. In recent years, the controversy as to whether hypoperfusion as a major cause of stroke is attributable to lacking of good standard of hemodynamic stroke and the interaction of hypoperfusion and embolism to cause ischemic stroke in many patients [14, 22]. In this study, we found that patients with ICA stenosis suffered from compromised global perfusion, compared with patients without ICA stenosis and the diminished perfusion was associated with the severity of ICA stenosis. In addition, our results found that stroke patients with unilateral stenosis had significantly more flow in the contralateral CCA than in the ipsilateral CCA, which reflects the existence of collateral blood flow to the supply territory of the ipsilateral CCA to maintain cerebral blood flow. Meanwhile, 25 patients had significant increase in BFV in ipsilateral CCA, implying that there were also pathways from inpsilateral ECA artery together with contralateral CCA to form the collateral circulation of stroke patients with carotid stenosis.

The most important finding of the present study was that CVIQ was able to assess the compensatory patterns of collateral flow from the contralateral ICA and bilateral VAs in stroke patients with unilateral carotid stenosis, indicating the presence of complete circle of Willis. While in patients with bilateral carotid stenosis, the option of supplying collateral flow through the anterior circle was insufficient, thus alternative collateral flow pathways such as the PCoAs became the most important route to contribute blood flow from the posterior circulation, which was consistent with previous MRA studies [23].

Our previous study revealed that CVIQ could accurately detect the presence of intracranial collateral circulation [8], while the present study demonstrates the cerebral blood flow redistribution from contralateral CCA and posterior circulation in patients with carotid stenosis, which reflects the compensating patterns and the presence of collateral pathways of patients with carotid occlusive disease, and illustrates that it is possible to quantify the effect of extracranial stenosis on distal cerebral circulation. What’s more, the presence of adequate compensating collateral flow may protect patients from the deterioration of clinical symptoms. Therefore, flow assessment may help decision making about patient selection for interventional therapy.

Compromised cerebral perfusion can be detected with various techniques, including positron emission tomography (PET), single photon emission computed tomography (SPECT), dynamic perfusion computed tomography (PCT), MRI dynamic susceptibility contrast (DSC), arterial spin labeling (ASL), and Transcranial Doppler [24]. Instead of using more popular technique to measure BFV to reflect cerebral perfusion, the present study opted to use CVIQ technique that has been validated in our laboratory, as CVIQ is more accurate in measuring cerebral blood flow volume than spectral Doppler technique and comparable to magnetic resonance angiography with phase contrast flow quantification [9, 10].

There are several limitations in our study. Firstly, the presence of intracranial artery stenosis was not analyzed because nearly 30 % recruited patients had no proper temporal window for TCD diagnosis. Subgroup analysis will be designed to evaluate the hemodynamic effects of intracranial artery lesions on the cerebral blood flow of collateral pathway by carotid ultrasonography in patients with carotid artery stenosis. We previously found that CCA and VA contribute maximally to collateral pathways in carotid occlusive diseases [8]. Secondly, the present study did not evaluate the association of compensating patterns and risk of recurrent stroke, while further study will indicate the prognostic effect of compensating patterns on patients with carotid stenosis that could be used to guide recommendations for interventional therapy.

## Conclusion

The present study demonstrates in stroke patients with unilateral severe carotid artery disease, contralateral carotid artery plays an important role rather than bilateral VAs in compensating reduced brain perfusion. Patients with bilateral carotid stenosis, however, fully rely on collateral flow via the posterior circulation.

## Abbreviations

ICA: internal carotid arteries
CVIQ: color velocity imaging quantification ultrasound
BFV: arterial blood flow volume
CCAs: common carotid arteries
VAs: bilateral vertebral arteries
SPECT: Single Photon Emission Computed Tomography
PWH: the Prince of Wales Hospital
TOAST: the Trial of ORG 10172 in Acute Stroke Treatment
PET: positron emission tomography
SPECT: single photon emission computed tomography
PCT: perfusion computed tomography
DSC: dynamic susceptibility contrast
ASL: arterial spin labeling
